# Body talk on social networking sites and appearance anxiety among college students: the mediating role of self-objectification and moderating role of gender

**DOI:** 10.3389/fpsyg.2025.1513923

**Published:** 2025-04-10

**Authors:** Jiayi Ruan, Ruicong Yu, Yunfeng Zhao, Lunfang Xie, Yaqi Mei

**Affiliations:** ^1^School of Nursing, Anhui Medical University, Hefei, China; ^2^School of Medicine, Southeast University, Nanjing, China; ^3^School of Health Management, Anhui Medical University, Hefei, China

**Keywords:** appearance anxiety, body talk, college students, mediating effect, self-objectification, social networking

## Abstract

**Introduction:**

Although the factors influencing appearance anxiety have been explored, the correlation and underlying mechanisms of action between social media body talk and appearance anxiety remain unclear. This study proposes research hypotheses and conducts mutual validation of scale data and real media data to explore the mechanism of action between variables.

**Methods:**

A mixed-methods design was employed: 512 college students completed questionnaires containing the Appearance Conversations with Friends Scale, the Appearance Anxiety Scale–Brief Version, and the Body Surveillance Subscale of the Objectified Body Consciousness Scale. Furthermore, 1,011 actual social media data entries were analyzed to complement the empirical framework.

**Results:**

The results showed that college students’ body talk on social networking sites, appearance anxiety, and self-objectification were significantly positively correlated. Body talk on social networking sites had a significant positive predictive effect on appearance anxiety, with self-objectification having a partial mediating effect. Gender played a significant moderating role between social media body talk and appearance anxiety (*β* = 0.53, *p* < 0.01), with male students’ social media body talk positively predicting appearance anxiety more significantly than female students (*β* = 0.52, *p* < 0.05).

**Discussion:**

These results extend objectification theory by demonstrating how technology-mediated body talk amplifies appearance anxiety through self-surveillance behaviors. These findings may be helpful for developing targeted interventions to reduce the risk of appearance anxiety among college students who often talk about their appearance on social networking sites.

## 1 Introduction

According to the 2018 China Youth Face Value Competitiveness Report, 98% of young people born in the 21st century compared their appearance to others before the age of 18 (Wang and Sun, 2023). Furthermore, with attractiveness of appearance playing an increasingly important role in social life, appearance anxiety has also become an increasingly serious social problem. Appearance display and appearance comparison have become important parts of college students’ social lives (Coelho et al., 2023); college students focus on physical appearance, emphasize the influence and social value of appearance, and talk about physical appearance in terms such as “how to achieve the ideal appearance,” thus ignoring the difference between the “real me” and the “ideal me.” Previous studies have found that psychiatric disorders, such as body dysmorphic disorder (Jordan et al., 2022), eating disorders (Brosof and Levinson, 2017), and social anxiety (Liao et al., 2023), have been associated with heightened concern with or negative perceptions of physical appearance. Therefore, it is of great practical significance to explore the generative mechanism of appearance anxiety and reduce its prevalence among college students.

With the advent of the digital society, China has made the development of the digital economy a high priority. Given that social networking sites (SNS) are now major channels of communication, body talk on these sites and appearance anxiety are inextricably linked. SNS body talk refers to mutual communication about physical appearance on social networks and is a specific way of using social media (Wang Y., 2023). College students are more likely than other age groups to prefer using social networks for communication; thus, online social networking has become one of the most important forms of social interaction among college students (Liu et al., 2018). The interactive features of social media, such as liking, commenting, or sharing, make body talk spread rapidly and generate a lot of discussion, and this is under algorithmic recommendation, body-related topics tend to have high appeal, making users participate in the discussion in a more aggregated manner (Wang et al., 2025). And the frequent focus on and discussion of idealized body image can lead to users’ dissatisfaction and anxiety about their own bodies, especially under the influence of social media’s comparative culture, where social comparative pressure affects their self-esteem and self-confidence, causing them to develop a negative, socially anxious state in real life (Abrante and Carballeira, 2023), and they may even resort to extreme dietary or exercise behaviors, such as over-dieting or over-exercising (Pfender et al., 2023). Therefore, this study proposes Hypothesis 1: Body talk on SNS is positively associated with appearance anxiety among college students.

In the process of communication, individuals often must look at themselves from an observer’s point of view to obtain a direct picture of how they look to the observer, a process known as self-objectification. Self-objectification, first proposed by Fredrickson and Roberts (1997), refers to the phenomenon of women examining their bodies from an observer’s perspective, treating their bodies more as objects to be viewed and evaluated on the basis of their physical appearance, and valuing their bodies in terms of physical appearance rather than ability. SNS body talk involves the publication of one’s appearance in the form of pictures or videos on social platforms, where peers, family members, and even strangers can comment. At the same time, content creators can also become commentators and observe the appearance characteristics of others on social platforms, thus forming self-surveillance and appearance comparison behaviors, where they evaluate their own appearance with a harsh eye (Reichenberger et al., 2022) and expect others to approve of their appearance (Kvardova et al., 2025). Frequent self-surveillance and comparison behavior often triggers an individual’s fear of negative evaluation of appearance, which can lead to negative emotions such as anxiety and depression (Jing and Jia, 2019). Accordingly, this study proposes Hypothesis 2: Body talk on SNS is positively associated with self-objectification, and self-objectification is positively associated with appearance anxiety among college students.

Indeed, viewing oneself as an object rather than a subject is self-objectifying behavior. This kind of self-objectification reflects an individual’s value bias- they are less concerned about developing their skills and more concerned about gaining recognition from others on the basis of their appearance (Chen et al., 2022). In Chinese society, where the availability of higher education is constantly expanding, the proportion of college students experiencing symptoms of anxiety and depression is significantly higher than that of the general population (Fu et al., 2020). The pressure exerted by academic studies and social interactions causes college students to desire an escape from reality. Therefore, they begin to seek a sense of belonging and spiritual fulfillment in cyberspace (Fabio and Iaconis, 2024), posting personal selfies and videos on SNS to obtain compliments from other users. This self-objectifying behavior allows creators to monitor the comments of others in real time, free from the constraints of the real world. However, as the number of posters increases, people increasingly compare their own appearance to that of others, becoming more concerned about whether their appearance meets social media esthetic standards and public expectations. Under the premise of this excessive body image comparison, the individual’s need to be recognized and praised will inevitably generate negative emotions (e.g., appearance anxiety) (Feng, 2023) when it is not satisfied. Accordingly, this study proposes Hypothesis 3: Self-objectification mediates the relationship between SNS body talk and appearance anxiety among college students.

Gender role theory (Jaehn et al., 2019) proposes that individuals of different genders differ in their attitudes and value orientations due to biological, psychological, and sociocultural influences. Research has shown that parallel social comparisons on youth’s willingness to undergo cosmetic surgery (Zhou et al., 2025), online communication on adolescent mental health (Birgisson et al., 2025), and emotional dissatisfaction on physical distress (López-Montón et al., 2024) are influenced by the moderating effects of gender. In addition, content (e.g., advertisements and fashion magazines) featuring an ideal male body image that is lean or muscular is becoming more prevalent in both traditional and social media, and that this body image is difficult to achieve in a natural way (De Jesus et al., 2015), further exacerbating men’s anxieties about their appearance and bodies. Therefore, this study proposes Hypothesis 4: Gender has a moderating effect on the relationship between social media body talk and appearance anxiety.

Robbins stress model (Robbins, 2009) analyzes individual stress response from the three links of “stress source - stress individual difference (stress experience) - stress result,” and the social comparison brought by body talk in social media serves as a source of stress, which makes the student group, facing the stereotyped and one-sided body image standard, involuntarily examine their own so-called “inadequacy” and generate serious self-objectification behavior and continue to generate appearance anxiety. “insufficiency,” resulting in serious self-objectification behaviors and continued derivation of appearance anxiety. The current study has a clear two-by-two relationship, but the evolution of the mechanism among the three is yet to be analyzed. In addition, the differences in family division of labor, social education and cognition between men and women in traditional Chinese culture have led to significant differences in behavioral responses under gender differences, which is in line with the “individual differences in stress.” In conclusion, based on the Robbins stress model, this study investigates the formation mechanism of appearance anxiety under the pressure of body talk in social media and examines whether gender plays a moderating role in the direct logic chain.

## 2 Materials and methods

### 2.1 Participants and procedure

Using the Wenjuanxing platform, a questionnaire survey was administered to college students at four medical colleges and universities in Anhui Province, China. The questionnaires were distributed online via WeChat, QQ, and other social platforms using convenience sampling methods. A total of 600 questionnaires were distributed. After excluding 88 invalid questionnaires (including logic errors, incomplete information, etc.), a total of 512 valid questionnaires were collected, with an effective response rate of 85.33%. Among the respondents, 104 (20.3%) were male, and 408 (79.7%) were female. The age of the respondents ranged from 17 to 25, with an average age of 20.16 ± 11.28. In light of the intricacies inherent in corporeal discourse, an online investigation was conducted on social media data, encompassing posts, comments, and interactions pertaining to body image. Topics related to body image are identified by reading the posts or comments, and keywords related to body image are extracted using natural language processing (LDA). Finally, the sentiment tendency of the posts or comments is analyzed using a sentiment analysis tool (SnowNLP).

### 2.2 Measurements

#### 2.2.1 SNS body talk

The Appearance Conversations with Friends Scale (Jones et al., 2004) modified by Wang et al. (2025) was used to measure each individual’s level of SNS body talk. Participants were asked to report their levels of SNS body talk on a 5-point Likert scale, where 1 = “never” and 5 = “very frequently,” with higher scores representing a higher frequency of SNS body talk. In this study, the Cronbach’s alpha for the scale was 0.893.

#### 2.2.2 Self-objectification

The Body Surveillance Subscale of the Objectified Body Consciousness Scale (Jackson and Chen, 2015) was used to assess the extent to which individuals were concerned about their physical appearance. Participants rated eight items on a 7-point scale, ranging from 1 = “not at all conforming” to 7 = “fully conforming,” with higher scores indicating more frequent physical monitoring, higher levels of body concern, and higher levels of self-objectification among the study participants. In this study, the Cronbach’s alpha for this scale was 0.781.

#### 2.2.3 Appearance anxiety

Appearance anxiety was assessed using the Appearance Anxiety Scale–Brief Version. Compiled by Dion et al. (1990) and translated and used by Chinese scholar Sun (2018), the scale was used to assess the level of appearance anxiety in the participants. Participants rated 14 items on a 5-point scale, ranging from 1 = “almost never” to 5 = “almost always” (e.g., “I am nervous about various aspects of my appearance,” “I am worried about how others evaluate my appearance”), with higher mean scores indicating higher levels of anxiety about an individual’s appearance. In this study, the Cronbach’s alpha for this scale was 0.862.

#### 2.2.4 Statistical analysis

The data analysis was conducted utilizing the SPSS 25.0 and PROCESS 3.5 software programs. Harman’s one-way method was employed to detect common method bias, while Pearson’s correlation analysis was implemented to elucidate variable correlations. The mediating and moderating effects were evaluated using Model 4 and Model 1 in PROCESS3.5, with 5,000 samples being simulated via the bootstrap method. The results were deemed to be significant if the 95% bias-corrected confidence interval did not contain 0, and the test criterion alpha was set at 0.05. In addition, the Bonferroni correction was used to correct for type I errors from multiple comparisons. The original significance level αin this study was set at 0.05 and three comparisons were made. Therefore, the corrected significance level αnew was 0.0167.

## 3 Results

### 3.1 Common method bias test

The common method bias test (Podsakoff et al., 2003) was performed using Harman’s single-factor test. The results showed that there were 10 factors with a characteristic root greater than 1; the amount of variance explained by the first factor was 21.24%, which was lower than the 40% critical threshold (Tang and Wen, 2020), indicating that common-method bias was not a serious problem in this study.

### 3.2 Descriptive statistics and correlation analysis

Table 1 presents the mean, standard deviation, and correlation matrix for each of the study variables. The correlation matrix table reveals significant positive correlations among SNS body talk, appearance anxiety, and self-objectification among college students. Specifically, the analysis revealed a correlation between SNS body talk and appearance anxiety, as well as between SNS body talk and self-objectification. Additionally, a correlation was found between appearance anxiety and self-objectification. Each correlation was statistically significant (*p* < 0.001).

**Table 1:** Descriptive statistics and correlation analysis between variables in the mediation model (*N* = 512).

Variables	*M*	SD	1	2	3
1. Body talk on social networking sites	11.89	4.19	1		
2. Appearance anxiety	39.85	8.43	0.330**	1	
3. Self-objectification	29.04	7.65	0.391**	0.533**	1

**p < 0.01.

Following the implementation of Pearson correlation analysis, *p*-values were obtained for multiple variables. To address the issue of multiple comparisons, each *p*-value was multiplied by the number of comparisons, *n* = 3, to obtain corrected *p*-values. The correlation between 3 variables remained significant after correction (*p*<0.003). The results of this analysis are presented in Table 2.

**Table 2:** Correlation between variables after Bonferroni Correction (*N* = 512).

Variable 1	Variable 2	Original *p*-value	Corrected *p*-value	Significance
SNS body talk	Appearance anxiety	<0.001	<0.003	Yes
SNS body talk	Self-objection	<0.001	<0.003	Yes
Appearance anxiety	Self-objection	<0.001	<0.003	Yes

### 3.3 Moderated mediation effects

**Figure 1:**
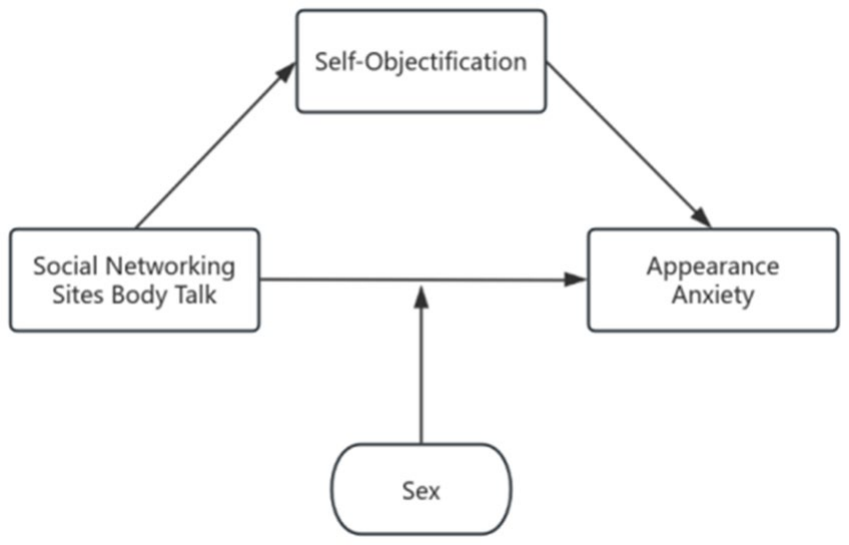
Plot of mediating effects of self-objectification in social media body talk and appearance anxiety and moderating effects of gender.

Prior to the analysis of the data, normalization of the scores for each factor was conducted. The mediated effects test was then executed using model 4 of PROCESS3.5. Model 1 demonstrated that social media body talk exhibited a positive predictive effect on appearance anxiety (*β* = 0.704, *p*<0.001). Model 2 further revealed that social media body talk had a positive predictive effect on self-objectification (*β* = 0.726, *p*<0.001). Model 3 indicated that following the incorporation of self-objectification, social media body talk still had a positive effect on appearance anxiety after the inclusion of self-objectification (*β* = 0.327, *p*<0.001). And self-objectification had a positive predictive effect on appearance anxiety (*β* = 0.519, *p*<0.001). Model 1 was further used to test the moderating role of gender in the relationship between social media body talk and appearance anxiety, and Model 4 demonstrated that the interaction term between social media body talk and gender had a positive predictive effect on appearance anxiety (*β* = 0.522, *p*<0.05), suggesting that different genders were able to modulate the positive effect of social media body talk on appearance anxiety, as illustrated in the Table 3 below. The mediation model was constructed by taking general demographic information as control variables, social media body talk as the independent variable, self-objectification as the mediator, and appearance anxiety as the dependent variable, and the mediation effect was tested by using the bootstrap method. The analysis was conducted using 5,000 simulated samples, and the results indicated that self-objectification mediated the relationship between social media body talk and appearance anxiety (see Table 4 and Figure 1).

**Table 3:** Regression analysis of mediation and moderation effects (*N* = 512).

Variable	Model 1 Appearance anxiety	Model 2 Self-objection	Model 3 Appearance anxiety	Model 4 Appearance anxiety
SNS body talk	0.704***	0.726***	0.327***	0.691***
Self-objection			0.519***	
Sex				0.521
SNS body talk*sex				0.522*
R^2^	0.3690	0.4051	0.5672	0.3828
ΔR^2^	0.1362	0.1641	0.3128	0.1466
F	15.9515	19.8724	39.9374	12.3647

All models incorporate confounding variables including whether the child is an only child, grade level, and monthly household income. *p < 0.05, ***p < 0.001.

**Table 4:** Mediation effect analysis of self-objectification (*N* = 512).

Effect	Effect value	Standard error	Bootstrap 95% CI		Relative effect value (%)
			Lower limit	Upper limit	
Direct effect	0.33	0.08	0.17	0.49	46.44
Mediation effect	0.38	0.05	0.28	0.48	53.56
Total effect	0.70	0.08	0.54	0.87	

### 3.4 Simple slope analysis

**Figure 2:**
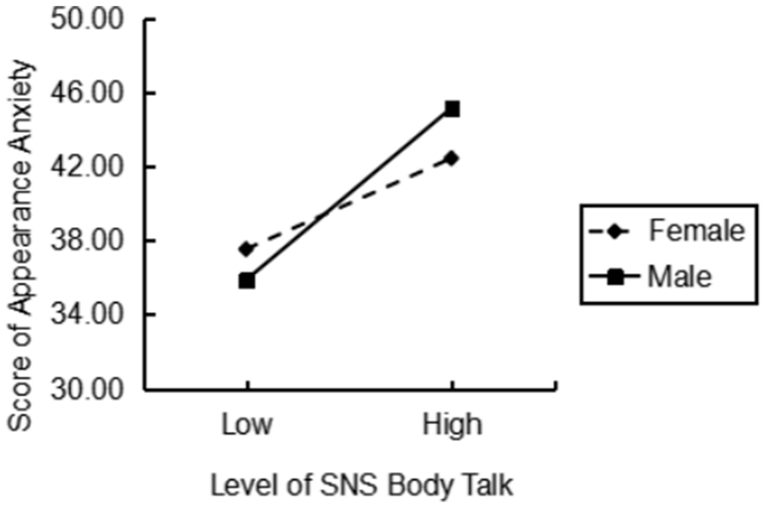
The role of gender in moderating the relationship between social media body talk and appearance anxiety.

Simple slope plots were drawn by taking one standard deviation each of positive and negative of social media body talk and appearance anxiety respectively, when the gender was female, social media body talk positively predicted appearance anxiety (*β* = 0.212, *p* < 0.05); when the gender was male, social media body talk positively predicted appearance anxiety (*β* = 0.750, *p* < 0.001), and it can be assumed that, compared with female, social media body talk positively predicts appearance anxiety more strongly in males (see Figure 2).

### 3.5 Actual social media data analysis

This study was conducted on the university student forums of two major Chinese social media platforms, Weibo and Xiaohongshu. By searching for keywords related to “body image,” a total of 1,011 valid data entries, including posts, comments, and interactions, were systematically collected and analyzed.

#### 3.5.1 Thematic analysis

A thematic analysis of 1,011 body image-related social media posts was conducted, yielding the following four predominant themes: appearance anxiety, body image management, appearance compliments and comparisons, and body confidence and self-acceptance.

#### 3.5.2 Sentiment analysis

**Figure 3:**
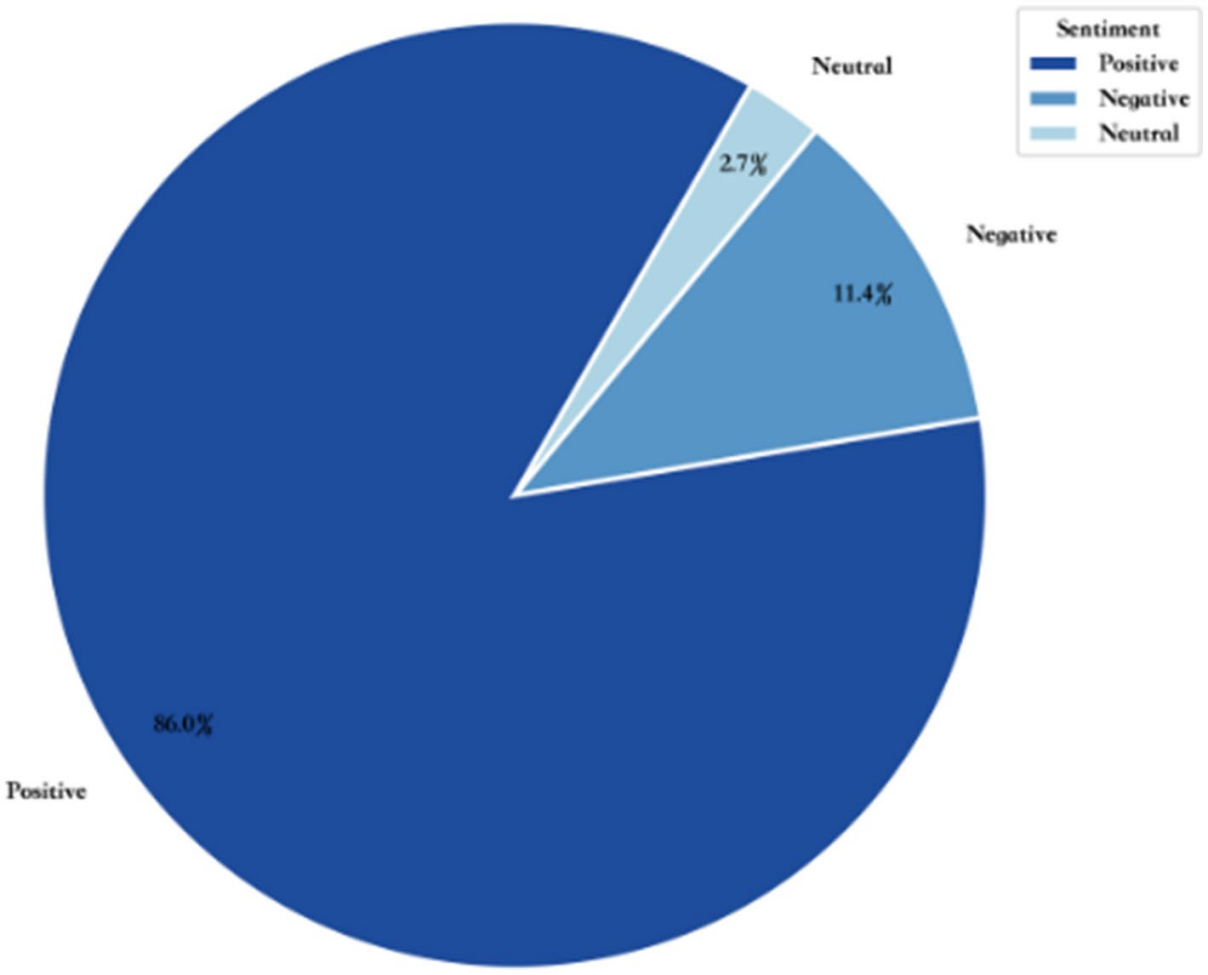
College student social media body talk emotion category pie chart.

The results of the sentiment analysis of 1,011 posts using the SnowNLP (Chinese sentiment analysis tool) are shown in Figure 3.

#### 3.5.3 Correlation analysis

In this study, likes, comments, and shares were employed as indicators of user engagement. After excluding posts with zero engagement across all three indicators, a total of 522 valid posts were included for analysis. Pearson correlation analysis revealed that the number of retweets of the same post was strongly positively correlated with the number of comments (*r* = 0.615, *p* < 0.01) and weakly positively correlated with the number of likes (*r* = 0.242, *p* < 0.01); the number of comments was also positively correlated with the number of likes (*r* = 0.195, *p* < 0.01). In addition, the frequency of words in the social media body talk category was significantly positively correlated with the frequency of words in the appearance anxiety category (*r* = 0.483, *p* < 0.01) and the frequency of words in the self-objectification category (*r* = 0.620, *p* < 0.01); the frequency of words in the self-objectification category was highly correlated with the frequency of words in the appearance anxiety category (*r* = 0.656, *p* < 0.01) (see Appendix 1).

## 4 Discussion

In recent years, China’s cosmetic industry has been experiencing a period of booming development (Sun, 2018), with the awakening of a sense of “beauty” sweeping across almost every industry. This has led to the rise of a variety of online shopping platforms and a “live-streaming marketing” culture. College students, one of the most active groups on social networks, have begun to pay closer attention to their appearance. However, a series of mental health problems has arisen in the process of comparing and being compared. To explore the generation mechanism of appearance anxiety among college students, we used an online questionnaire to investigate their appearance anxiety, aiming to provide a reference for subsequent intervention and adjustment measures.

The research findings showed that SNS body talk had a significant positive predictive effect on appearance anxiety, and Hypothesis 1 was verified. Namely, the higher the frequency of SNS body talk, the higher the level of individual appearance anxiety, which is consistent with Chinese research (Wang Y. J., 2023) and international research (Walker et al., 2015). Online socialization lacks the proximity and familiarity of traditional offline socialization, and the information it conveys is characterized by arbitrariness, editability, and wide dissemination. Therefore, the images used to display appearance on SNS are likely to be edited and modified by creators to attract others’ “likes” and to display the ideal perfect self-image. Women’s beauty is often depicted in society as conforming to an “ideal thinness,” while men’s attractiveness is frequently portrayed as adhering to an “ideal muscular body.” Over time, this mainstream esthetic has evolved toward a standardized perception, wherein certain body types are promoted as the norm. This standardization process involves the gradual reinforcement and dissemination of these ideals across various media platforms and cultural contexts. At the same time, some college students may lack the ability to think rationally and independently, and are easily influenced by this type of normatively attractive content, subsequently developing a dependence on social media (Wangqu et al., 2024) under such a premise, they often reveal a widening gap between themselves and mainstream esthetics in the process of talking about their bodies, which leads to negative emotions (e.g., appearance anxiety and low self-esteem) and has an extremely negative impact on their mental health and quality of life (Hooper et al., 2023).

Our results also showed that SNS body talk predicted self-objectification, and self-objectification predicted appearance anxiety, thus validating Hypothesis 2. SNS users constantly upload personal images to their home pages, view likes and comments as recognition of their “*yangzhi*” (a Chinese term that combines the characters for “face” and “value”) by others, and indulge in the illusion of beauty. This process creates commodities for the self and others to “gaze” at (Zhou, 2023), taking the attribute of appearance as the most important criterion for measuring one’s value, and viewing oneself from the perspective of an observer (i.e., self-objectification). Images enhanced by beauty technology retain the physical attributes of the human body, effectively serving as an extension of one’s physical self. Individuals tend to associate with the transformed appearance depicted in these images (Xu, 2023). Over time, when college students stop “fine-tuning” their appearance online and return to reality, they will also scrutinize unbeautified pictures of themselves, worrying about whether others would recognize their appearance in reality, thus aggravating their appearance anxiety.

Our results suggest that self-objectification plays a partially mediating role in the influence of SNS body talk on appearance anxiety, thus validating Hypothesis 3. Frequent participation in SNS body talk leads to a deepening of self-objectification, and individuals with high levels of self-objectification use appearance as a measure of their own value, aspire to meet harsh esthetic standards, and fear negative evaluations, resulting in appearance anxiety. There are also many influencing factors mediating between SNS body talk and appearance anxiety, such as interpersonal sensitivity and body esteem (Maftei, 2022). Thus, self-objectification can mediate the process of SNS body talk leading to appearance anxiety, but it is not the only mediator. The inability of people of different genders, ages, and cultural backgrounds to maintain complete consistency in their appearance highlights individual uniqueness, and this suggests that social media should reject comparative body talk and create a positive social atmosphere. College students can improve their ability to cope with stressful life events through positive meditation training (Gao et al., 2023). At the same time, they should pay attention to the development of diverse esthetic norms, recognizing and accepting the self without considering appearance as their most important attribute.

The findings of the present study indicated that gender played a significant moderating role in social media body talk and appearance anxiety, thus validating Hypothesis 4. While typically women show higher rates and more pronounced symptoms of appearance anxiety, men may show stronger appearance anxiety in certain specific contexts. Research suggests (Liu, 2023) that men tend to downwardly compare and have higher levels of body esteem (Pan and Peña, 2019), and evolutionary psychology states that men’s self-confidence is more susceptible to feedback signals from others (Buss, 1988). Consequently, men are more concerned about comments from the opposite sex than comments from the same sex, and are more likely to make the lowest social comparisons, feel the lowest motivational emotions, and the highest negative emotions, especially when faced with unattainable comments from the opposite sex (Pan and Mu, 2024). In contrast, women who tend to make upward comparisons receive social media talk about ideal body image and instead believe they can achieve their goals and take action (Buote et al., 2011).

The results of 1,011 analyses of actual social media data support the conclusions drawn from the scale analysis. The strong correlation between the frequency of appearance anxiety words and the frequency of self-objectification words suggests that users’ frequent scrutiny of their appearance through social media (e.g., using beauty filters, engaging in body image comparisons) may exacerbate the tendency to perceive the body as an object to be evaluated, which in turn induces anxiety. This is consistent with Brasil’s findings (Brasil et al., 2024) that technology-mediated body surveillance behaviors deepen the perceived severance between real and ideal appearance. Posts involving body image were more likely to trigger comment interactions, suggesting that users tend to confirm the value of appearance through feedback from others, a process that further reinforces users’ self-objectification and creates a vicious cycle of “discussion-anxiety-more discussion.”

## 5 Conclusion

The results of this study revealed the formation mechanism of appearance anxiety in college student groups, highlighting the influence path between SNS body talk and self-objectification. The richness of social media helps college students to improve their communication and interaction skills to a certain extent. However, nonessential appearance anxiety in the pursuit of ideal beauty can easily lead to high levels of self-objectification and negative emotions in college students.

### 5.1 Limitations

This study was limited in the following aspects. Firstly, this study utilized a cross-sectional survey, which did not allow for causal conclusions to be drawn. Experimental designs can be incorporated into future studies to enhance the causal interpretation of the results. Secondly, it did not examine posting frequency or individual psychological data, which are critical for understanding the underlying mechanisms. Future research should incorporate these elements and employ surveys to establish causal relationships. Finally, the sample data of this study had a high proportion of females, which may produce result bias, and subsequent studies need to further optimize the sample structure to reduce the occurrence of such bias.

## Data Availability

The raw data supporting the conclusions of this article will be made available by the authors, without undue reservation.
