# Supplementary material for: Body talk on social networking sites and appearance anxiety among college students: the mediating role of self-objectification and moderating role of gender
Source: Front Psychol. 2025 Apr 10;16:1513923. doi: 10.3389/fpsyg.2025.1513923 (PMC12020388; doi:10.3389/fpsyg.2025.1513923)
Supplement: Supplementary file 1 [file Table_1.docx]

Appendix 1

In our study, the hypothesis of the influence of social media body talk and self-objectification was proposed for college students' appearance anxiety, and data on the actual state of college students were collected through a subjective scale for the purpose of verifying the hypothesis. The data results showed that body talk on social networking sites had a significant positive predictive effect on appearance anxiety, with self-objectification having a partial mediating effect.

In order to achieve mutual complementation and validation between the actual social media data and the scale, our study attempted to carry out the analysis through two methods:

Method 1 (Post-Level Analysis):

We extracted interaction metrics (likes, comments, retweets) and keyword frequencies (appearance anxiety-related, body talk-related, self-objectification-related) for each post and calculated their correlations.

Method 2 (User-Level Analysis):

We initially planned to link posting frequency with psychological variables using user IDs. However, due to data limitations (public Weibo data lacks continuous user posting behavior and individual psychological measurements), this method was not feasible.

Therefore,we adopted Method 1 and refined the dataset:

Original data included 1,011 posts, of which 489 had zero interaction metrics (likes, comments, retweets), likely representing invalid or low-engagement content (e.g., ads, system-generated posts).

Therefore, zero-interaction posts were excluded, retaining 522 valid posts for correlation analysis to ensure statistical robustness (zero-interaction posts may not reflect genuine user behavior).

Analysis of the 522 valid posts revealed:

1. Interaction Metric Correlations(as shown in table 1):

Retweets strongly correlated with comments (r = 0.615, p < 0.01) and weakly with likes (r = 0.242, p < 0.01), suggesting high-repost content fosters deeper discussion.Comments also correlated with likes (r = 0.195, p < 0.01), suggesting such type of topic has a high level of heat and attention.

# Supplementary Figures and Tables

- **Table 1:**

| *Pearson correlation value of between post likes in relation to comments and retweets（N=522）* | | | | | |
| --- | --- | --- | --- | --- | --- |
|  | M | SD | Likes | Comments | Retweets |
| Likes | 1567.4 | 8854.23 | 1 | - | - |
| Comments | 123.29 | 503.12 | .195** | 1 | - |
| Retweets | 83.00 | 455.13 | .242** | .615** | 1 |
| *** p ≤ .001, ** p < .01, * p < .05 | | | | | |

1. Body Image and Psychological Variables(as shown in table 2):

Body talk keywords strongly correlated with Appearance anxiety keyword frequency (r = 0.483, p < 0.01) and self-objectification keywords (r = 0.620, p < 0.01).Self-objectification keywords significantly correlated with Appearance anxiety keyword frequency (r = 0.656, p < 0.01).

- **Table 2:**

| *Pearson correlation value of between Body talk keywords frequency in relation to Self-objection*  *keywords frequency and Appearance anxiety keyword frequency（N=522）* | | | | | |
| --- | --- | --- | --- | --- | --- |
|  | M | SD | Body talk keywords frequency | Self-objection keywords frequency | Appearance anxiety keyword frequency |
| Body talk keywords frequency | 1.38 | 1.46 | 1 | - | - |
| Self-objection keywords frequency | 1.33 | 1.61 | .620** | 1 | - |
| Appearance anxiety keyword frequency | 1.90 | 1.63 | .483** | .656** | 1 |
| *** p ≤ .001, ** p < .01, * p < .05 | | | | | |

1. Comments weakly correlated with appearance anxiety (r = 0.091, p < 0.05) and self-objectification keywords (r = 0.136, p < 0.01), indicating posts involving self-monitoring may trigger more engagement.

The above results are consistent with the results of the analysis of the scale data in this study.
